# Use of inverse modeling to evaluate CENTURY-predictions for soil carbon sequestration in US rain-fed corn production systems

**DOI:** 10.1371/journal.pone.0172861

**Published:** 2017-02-24

**Authors:** Hoyoung Kwon, Carmen M. Ugarte, Stephen M. Ogle, Stephen A. Williams, Michelle M. Wander

**Affiliations:** 1 Environment and Production Technology Division, International Food Policy Research Institute, Washington, DC, United States of America; 2 Department of Natural Resources and Environmental Sciences, University of Illinois, Urbana, IL, United States of America; 3 Natural Resource Ecology Laboratory, Colorado State University, Fort Collins, CO, United States of America; University of Oregon, UNITED STATES

## Abstract

We evaluated the accuracy and precision of the CENTURY soil organic matter model for predicting soil organic carbon (SOC) sequestration under rainfed corn-based cropping systems in the US. This was achieved by inversely modeling long-term SOC data obtained from 10 experimental sites where corn, soybean, or wheat were grown with a range of tillage, fertilization, and organic matter additions. Inverse modeling was accomplished using a surrogate model for CENTURY’s SOC dynamics sub-model wherein mass balance and decomposition kinetics equations from CENTURY are coded and solved by using a nonlinear regression routine of a standard statistical software package. With this approach we generated statistics of CENTURY parameters that are associated with the effects of N fertilization and organic amendment on SOC decay, which are not as well quantified as those of tillage, and initial status of SOC. The results showed that the fit between simulated and observed SOC prior to inverse modeling (*R*^*2*^ = 0.41) can be improved to *R*^*2*^ = 0.84 mainly by increasing the rate of SOC decay up to 1.5 fold for the year in which N fertilizer application rates are over 200 kg N ha^-1^. We also observed positive relationships between C inputs and the rate of SOC decay, indicating that the structure of CENTURY, and therefore model accuracy, could be improved by representing SOC decay as Michaelis-Menten kinetics rather than first-order kinetics. Finally, calibration of initial status of SOC against observed levels allowed us to account for site history, confirming that values should be adjusted to account for soil condition during model initialization. Future research should apply this inverse modeling approach to explore how C input rates and N abundance interact to alter SOC decay rates using C inputs made in various forms over a wider range of rates.

## Introduction

**Fig 1:**
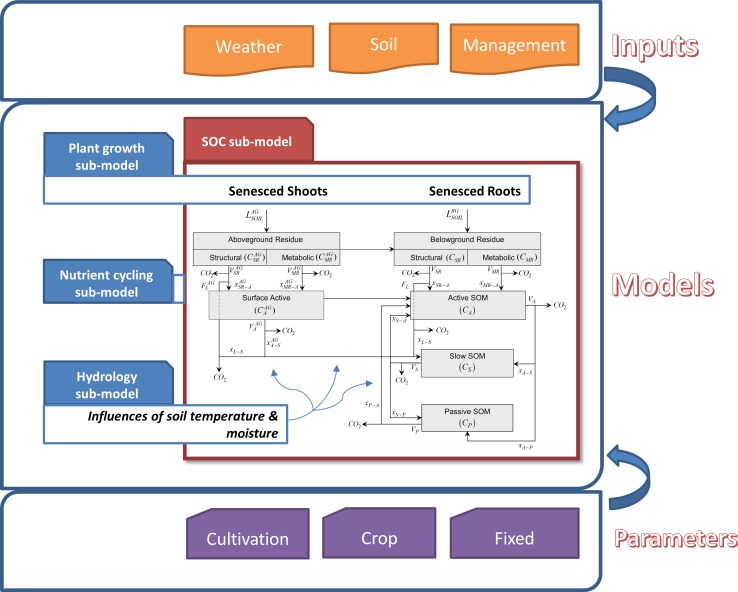
The environment and sub-models of CENTURY soil organic matter model.

The CENTURY soil organic matter (SOM) model [1] is an agro-ecosystem model developed to simulate the dynamics of multiple SOM “compartments” or “pools”, which differ in their size and degree of physical and/or chemical stabilization, under various agronomic practices and soil/climatic conditions (Fig 1).

The model has been widely used to guide soil-based nutrient management, mitigate agricultural non-point source pollution, and promote soil organic carbon (SOC) sequestration. Importantly, CENTURY and closely associated models are being used for accounting of national inventories and policy development [2–5]; this highlights the need for continual model refinement resulting from improved understanding of factors influencing model kinetics.

Like other process models describing SOM dynamics, CENTURY’s performance depends on the accuracy of parameter values employed and the degree to which the initial status of the modeled system is known. Accuracy and precision of predictions are generally improved for site-specific application with model calibration wherein the model’s initial conditions and parameter values are adjusted for a site or region against observed data. CENTURY calibration typically includes adjustment of i) the initial distribution of SOC among three SOM pools (active, slow, and passive) and ii) one or more of the parameters related with decay rates of those pools [6, 7]. This type of calibration usually involves manual adjustments, which result in variable success and uncertainties in model prediction that partly depend on the modeler’s experience [8].

While systematic approaches such as automatic calibration algorithms can be used to overcome the subjectivity inherent in “by-hand” approaches to calibration, statistical methods can be used to quantify model uncertainty. For example, Yeluripati et al. [9] used a Bayesian approach to reduce and quantify the uncertainty in the initial conditions of the daily time step version of CENTURY. More recently, Kwon and Hudson [10] adapted an inverse modeling approach using a surrogate model for CENTURY’s SOC dynamics sub-model (SCSOC). A nonlinear regression routine of a standard statistical software package was used to provide a statistical perspective on the parameters and their influence on estimates of SOC computed by CENTURY.

Using the SCSOC on long-term data collected from conventionally tilled corn-based systems at the Morrow Plots in Illinois, Kwon and Hudson [10] found that CENTURY overestimated the positive effects of N fertilization on SOC. This effect was attributed to overestimation of the positive effects of C inputs on SOC levels and under-estimation of the stimulatory effects of N addition on decay rates. Results presented by Ogle et al. [11] suggested that CENTURY-modeled SOC is overestimated in N fertilized systems, and agreed with the conclusion of Kwon and Hudson [10]. In their work, a linear mixed effect model was used to quantify the accuracy and precision in modeled SOC using data from 47 agricultural experiments selected to represent the most prevalent cultivation methods in US row crop systems. A similar approach was applied by Ogle et al. [12] to demonstrate that the model structure and parameterization are the largest sources of uncertainty associated with CENTURY based estimates of SOC. Recent work by Fujita et al. [13] showed that SOM decomposition models can be improved by considering interactions between C and N inputs and their effect on decay rates and stock flows. Another source of poor model performance is inaccurate prediction of C inputs [14]. Uncertainty associated with model-based estimates of C inputs, which are greater than those associated with N inputs in fertilized systems, must be better quantified.

In this study, we used SCSOC to evaluate the structure and parameterization of CENTURY’s SOC sub-model using field measured data obtained from multiple crop production sites. Datasets like the one developed by Ogle et al. [11] are ideal for parameter calibration using inverse modeling as they provide information about changes in organic matter stocks associated with management applied across a large environmental gradient. In addition to allowing us to obtain more precise and accurate parameter estimates, these data allow us to directly evaluate the influences of key factors including C inputs, yield, and N inputs, on SOC decay rates [15]. We inversely modeled SOC data to estimate CENTURY parameters reflecting the i) influence of yield and associated estimates of litter inputs to soil, ii) initial status of SOC, and iii) effects of management, mainly N fertilization and organic amendments, on SOC decay kinetics.

## Materials and methods

### Overview of SCSOC model structure and inverse modeling

**Fig 2:**
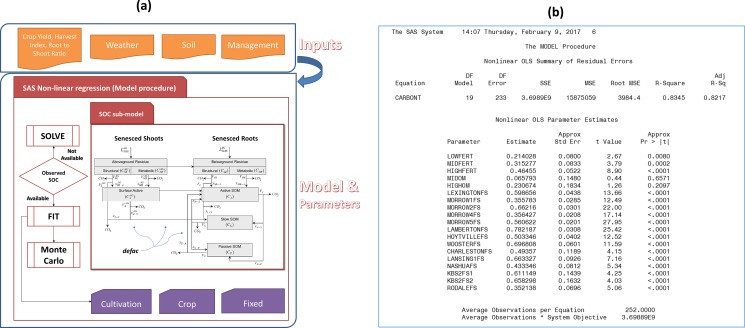
Overview of the surrogate CENTURY’s SOC dynamics sub-model (SCSOC). (a) SCSOC’s structure, data inputs, and nonlinear regression (Model) procedure performed in SAS statistical software and (b) summary statistics generated by the SCSOC run.

The SCSOC was developed as a nonlinear regression tool to rapidly and objectively optimize site-specific parameters for CENTURY using time-series data [10] (Fig 2A).

It is coded within the Model procedure of SAS [16] to provide parameter estimation, simulation, and forecasting of dynamic nonlinear simultaneous equation models. The SCSOC represents the same conceptual pools as the CENTURY’s SOC sub-model, including i) aboveground and belowground plant litter (residues), which are divided into structural (*SR*) and metabolic (*MR*) litter pools depending on their lignin content, ii) active microbes in surface residues, and iii) active (*A*), slow (*S*) and passive (*P*) SOM pools. The temporal evolution of each pool *j* (*C*_*j*_(*t*)) is expressed by combining the rate expressions for decomposition (Eq 1) with the resultant product formation rates and litter input fluxes to form a set of coupled, differential mass balance equations describing each plant litter and SOM pool.

$$\frac{dC_{j}(t)}{dt}=\left[x_{MR,j}\cdot v_{MR}\right]+\left[x_{SR,j}\cdot v_{SR}\right]+\left[x_{A,j}\cdot v_{A}\right]+\left[x_{S,j}\cdot v_{S}\right]+\left[x_{P,j}\cdot v_{P}\right]-v_{j}$$ (1)

where *x*_*MR*,*j*_ is the fraction of the decomposed C derived from the metabolic litter C pool that flows into the SOM pool *j* and *v*_*MR*_ is the decomposition rate of the metabolic litter C pool. The same convention and notion is applied to other SOM pools.

In CENTURY, these equations are numerically solved for a series of fixed time intervals using a first-order numerical procedure (Euler method) that approximates the decomposition rate over an entire month, *v*_*j*_(*m*), using *C*_*j*_(*m*), which denotes the value of C at the beginning of the month (Eq 2):

$$v_{j}(m)=k_{j}^{*}(m)\cdot C_{j}(m)$$ (2)

where $k_{j}^{*}(m)$ is a site-specific decay rate coefficient for SOM pool *j* and its value is the product of the maximum decay rate coefficient in uncultivated soil (S1 Table) and factors that account for i) the site-specific effects of residue quality and soil texture, ii) temporally variable influences of soil temperature and moisture (*defac*), and iii) soil tillage effect (*clteff*).

Instead of using the Euler method, the SCSOC employs the Crank-Nicholson method, which uses the average of *C*_*j*_(*m*) and *C*_*j*_(*m*+1) to solve the differential equations with SAS’ Model procedure (Eq 3).

$$v_{j}(m)=k_{j}^{*}(m)\cdot \frac{C_{j}(m+1)+C_{j}(m)}{2}$$ (3)

Using the Model procedure, the SCSOC can perform an inverse modeling of observed SOC data to estimate CENTURY parameters and their standard errors (SE). It can also test for statistical significance and be used to rapidly and efficiently conduct uncertainty analysis using Monte Carlo (MC) simulation.

Importantly the SCSOC allows users to derive time-dependent variables, which are typically simulated in plant growth, nutrient cycling, and hydrology sub-models of CENTURY (Fig 1), from model input datasets. As a result, it can isolate and quantify individual sources of uncertainty associated with modeling SOC dynamics. For example, by decoupling C and N sub-models it can empirically evaluate the effects of N inputs on SOC decay.

Key model variables—aboveground and belowground residues replaced by crop/plant C input rates to soil ($L_{SOIL}^{AG}$ and $L_{SOIL}^{BG}$) and climatic factors represented by the *defac* parameter—are read into the SCSOC from model input datasets (Fig 2A). While the C input rates are empirically estimated using productivity data and relationships between yield or aboveground biomass and agronomic indices (yield-based C inputs) [i.e. the ratio of grain mass to total aboveground plant production (harvest index, HI), and the ratio of total aboveground shoot production to total root production (root to shoot ratio, RSR)], they can be directly derived from the plant growth routine in CENTURY (CENTURY-modeled C inputs) and used in the SCSOC. Note that CENTURY-modeled C inputs include feedbacks from the CENTURY’s N sub-model where the empirical approach reflects soil N (and weather-based) impacts on plant growth in achieved yields. Like $L_{SOIL}^{AG}$ and $L_{SOIL}^{BG}$, *defac* can be directly derived from empirical fits as well.

### Application of SCSOC inverse modeling to US rainfed corn production systems

We used data from 10 sites described in Ogle et al [11] to evaluate the CENTURY predictions for SOC sequestration using the SCSOC. These were selected from among 47 sites because yield data was recorded during the experimental periods and at least two SOC or SOM observations in top soil (soil depths of 0–20 cm) had been made over time (Table 1).

**Table 1:** Summary of key information on the sites used for CENTURY calibration using the SCSOC.

Location	Experiment start	Texture (Clay/Sand)	Prior land use	Annual average precipitation	Annual average temperature	Treatment^a^		Number of SOC observations	Simulation period and duration
						Number of treatment			
	‒ Year ‒	‒ Fraction ‒		‒ mm ‒	‒ °C ‒	‒ # ‒	‒ Details^b^ ‒	‒ # ‒	‒ yrs ‒
Lexington, KY	1970	0.29 /0.07	Bluegrass pasture (~50 yrs)	1140	13	8	1 crop rotation (CC) × 4 N rates (0, 84, 164, and 336) × 2 tillage (CT and NT)	24	1970–1990
Urbana, IL	1876	0.24 /0.09	Tall grass prairie	938	11.1	8	2 crop rotations (CC and CS) × (3 N rates (0, 224, and 336) + 1 OM (4.5 Mg ha yr^-1^)) × 1 tillage (CT)	172	1955–1992
Lamberton, MN	1960	0.27 /0.31	Tall grass prairie	632	6.2	4	1 crop rotation (CC) × 4 N rates (0, 40, 80, and 160) × 1 tillage (CT)	8	1960–1992
Hoytville, OH	1963	0.40 /0.21	Cultivation (6 yrs)	845	9.5	6	3 crop rotations (CC, CS, & SC) × 1 N rate (200) × 2 tillage (CT and & NT)	12	1963–1992
Wooster, OH	1962	0.15 /0.25	Grass meadow (6 yrs)	905	9.1	6	3 crop rotations (CC, CS, & SC) × 1 N rate (250) × 2 tillage (CT and & NT)	13	1962–1992
South Charleston, OH	1963	0.20 /0.15	Cultivation (6 yrs)	952	11.9	2	1 crop rotation (CC) × 1 N rate (250) × 2 tillage (CT and & NT)	4	1963–1992
East Lansing, MI	1963	0.10 /0.80	Pasture and cultivation	782	8.6	5	1 crop rotation (CC) × 1 tillage (CT) × (2 N rates (0 and180) + 3 OM (6, 12, and 18 Mg ha yr^-1^))	20	1968–1982
Nashua, IA	1978	0.22 /0.32	Cultivation	825	7.3	6	3 crop rotations (CC, CS, and SC) × 2 tillage (CT and NT) × 1 N rate (180)	13	1978–1989
KBS, MI	1980	0.18 /0.40	Cultivation	632	8.4	2	1 crop rotation (CSW) × 1 N rate (150) × 2 tillage (CT and NT)	24	1989–2001
Rodale, PA	1981	0.30 /0.17	Pasture and cultivation	1045	12.4	3	1 crop rotation (CS) × 1 N rate (150) × 1 tillage (CT) × triplicates	8	1981–1992

^a^ All sites with N treatment and tillage while two sites with organic matter additions.

^b^ Crop rotation: CC, continuous corn; CS, corn-soybean; CSW, corn-soybean-wheat; SC, soybean-corn. Tillage: CT, conventional tillage; NT, no tillage. The unit of N rate is kg N ha yr^-1^.

The dataset includes detailed information about management practices, monthly temperature and precipitation, soil textural classes, grain yields, and SOC or SOM levels (Table 1). By using the dataset to parameterize CENTURY, Ogle et al. [11] ran CENTURY simulations and generated output files that contained CENTURY- modeled values of the $L_{SOIL}^{AG}$, $L_{SOIL}^{BG}$, *defac* and losses of SOC resulting from pre-agricultural and agricultural periods of interest. We used those CENTURY output files to derive monthly C input rates and *defac* variables.

The initial fraction of SOC in the slow SOM pool (*f*_*S*_) and the management effect factor (*mgmteff*), which accounts for the combined effects of tillage, N fertilization, and organic matter (OM) additions on SOC decay of either the active or slow SOM pool, were estimated using the SCSOC fit to observed SOC or SOM data. Model parameters that were not estimated through calibration procedures or treated as input variables used CENTURY default values. We inversely estimated *f*_*S*_ at each of the 10 experimental sites and multiplied them with SOC reported at the beginning of each simulation to assign SOC masses to each of the three pools. In cases where initial SOC observations varied among treatments within a site, we estimated *f*_*S*_ for individual treatments. The *f*_*A*_, which is normally small (0.02 ~ 0.05) and rapidly adjusts to residue input rates [10], was assumed to be 0.02 regardless of treatment or site and *f*_*P*_ was set to make the fractions sum to unity. This resulted in estimation of a total of 14 parameters related to initial SOC status.

To estimate *mgmteff*, we first defined it as a product of the effects of three management practices and their interactions in our calibration.

mgmteff=clteff⋅ferteff⋅omeff (4)

Where *clteff*, *ferteff*, and *omeff* are the effects of tillage, N fertilization, and OM addition (e.g. manure application) on SOC decay. These variables take on values of unity for experimental treatments and years when the practices were not in effect and assume a fitted value where and when these practices were applied. To allow us to follow up on previous work suggesting that the effects of fertilization might be improved by calibration and reduce the confounding effects resulting from simultaneous calibration of three management effects, we fixed the *clteff* effect to a value of 1.4 applied over the year following tillage to achieve the effects of CENTURY’s default value of 5.5 applied for the month in which moldboard plowing or conventional tillage occurs before returning to unity for the other 11 months. To estimate *ferteff* and *omeff*, we classified studies according to levels of N fertilization [low (≤100), mid (100~200), and high (≥200) kg N ha^-1^ rates] and OM addition [mid (≤10) and high (≥10) t dry matter ha^-1^ rates] and assumed coefficients would be greater than zero, which allowed them to be log-transformed to estimate *λ*_*FERT*_ and *λ*_*OM*_ which equal *ferteff* and *omeff* after exponentiation:

$$\begin{array}{l} ferteff=e^{\lambda_{FERT}}\\ omeff=e^{\lambda_{OM}} \end{array}$$ (5)

Note that when *λ*_*FERT*_ and *λ*_*OM*_ are not significantly different from zero, *ferteff* and *omeff* equal one.

Finally, we ran the model to inversely estimate SOC and to generate the summary statistics of nonlinear ordinary least square parameter estimates (Fig 2B).

For the parameters of management effects, we reported the estimates after the log-transformation.

### Sensitivity analyses of model inputs

Comparison of C input rates derived from observed yields using agronomic indices and modeled yields allowed us to determine how sensitive parameter estimates were to C input rates. Observed grain yields, HI [17–21], and RSR [22] were used in Eqs 6 and 7 to determine input rates.

$$L_{SOIL}^{AG}=Y\cdot (100-\theta )\cdot C^{AG}\cdot \frac{\left(1-HI\right)}{HI}\cdot f^{MIX}$$ (6)

$$L_{SOIL}^{BG}=Y\cdot (100-\theta )\cdot C^{BG}\cdot \frac{\left(RSR\right)}{HI}\cdot RD$$ (7)

Where *Y* is yield (Mg ha^-1^), *θ* is the moisture content in grain (%), *C*^*AG*^ and *C*^*BG*^ are concentration of C in either aboveground or belowground litter (Mg C kg dry matter^-1^) [23–24], and *RD* is the fraction of root mass at certain soil depth (Table 2).

**Table 2:** Literature-derived values for agronomic indices and crop C composition used to compute C returned to soils.

	Harvest index (HI)	Root to shoot ratio (RSR)	A fraction of root mass in 0–20 cm soil depth (RD)	Moisture content in grain	C concentration of crop residue	
					Above-ground	Below-ground
				^___^ % ^___^	^___^ kg C kg dry matter^-1___^	
**Corn**	0.53	0.55	0.80	15	0.437	0.343
**Soybean**	0.46	0.56	0.80	13	0.454	0.467
**Winter wheat**	0.45	0.62	0.90	14	0.370	0.296
**Manure**					0.450	

We assumed that conventional tillage (CT) and no tillage (NT) differ in the fractions of aboveground litter that is transferred to soils (*f*^*MIX*^), with values of 0.95 and 0.05 being added, respectively according to CENTURY default values. The C inputs added as OM were similarly calculated by modifying Eq 6.

### Uncertainty analyses of model predictions

In addition to parameter estimation, we conducted an uncertainty analysis of model predictions. For this, we ran 1,000 MC simulations by employing parameter estimates along with their approximate standard errors and using covariance matrices obtained from inverse modeling. We used the MC simulations to compute distributions of model predictions and associated confidence limits. We also investigated how uncertainty in observations affects model predictions by generating “synthetic” observed SOC data that are randomly drawn from a normal distribution having a mean as an observed SOC data and a standard deviation that represent of 10% of the observed SOC. While a 10% uncertainty level may be lower than that associated with measurements taken at some individual sites, this level of uncertainty is high compared to the “minimum detectable difference” that has been suggested for SOC change considered by greenhouse gas registries and C trading mechanisms over a 5-yr period [25–28]. Similarly, “synthetic” yield data were also generated using the same approach as for SOC. Using these “synthetic” observed SOC and yield datasets, we conducted inverse modeling, followed by 1,000 MC simulations. We assumed that the confidence interval of model predictions constructed from the MC simulations would reflect the uncertainties of model inputs, parameters and structure.

## Results and discussion

### Parameter estimates and C input rates

**Fig 3:**
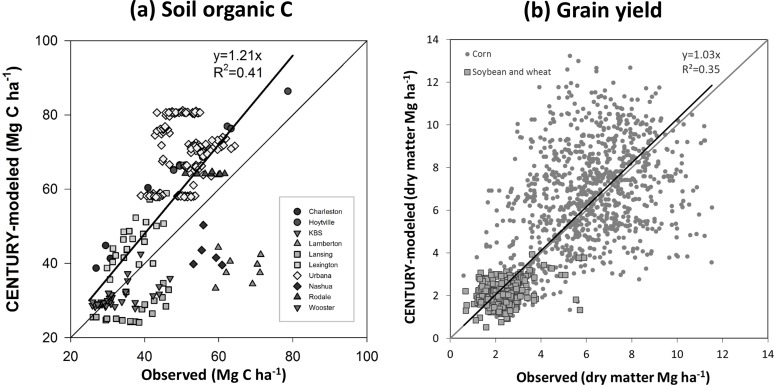
Comparison of (a) CENTURY-modeled soil organic C with observed soil organic C and (b) CENTURY-modeled grain yields with observed grain yields. The grey lines are 1:1 lines while the black lines are regression lines. All *R*^*2*^ are significant at *P*<0.05.

Prior to inverse modeling, we evaluated the fit between observed SOC at experimental sites and CENTURY-modeled SOC from the dataset and output files compiled by Ogle et al. [11] (Fig 3A), which revealed a less than optimal fit (Fig 3A; *R*^*2*^ = 0.41, *P*<0.05), as is typical for uncalibrated studies [e.g. 14].

This is partly explained by the inclusion of simulated yield achieved by a mixture of crops and management systems in the regression. While simulated and measured yields were positively related when all systems were considered together (Fig 3B), the inclusion of yields for fertilized and unfertilized corn and soybean/wheat resulted in considerable spread for individual crop types such that the correlation between simulated and measured yields for individual crops (corn or soybean/wheat) was not significant.

**Fig 4:**
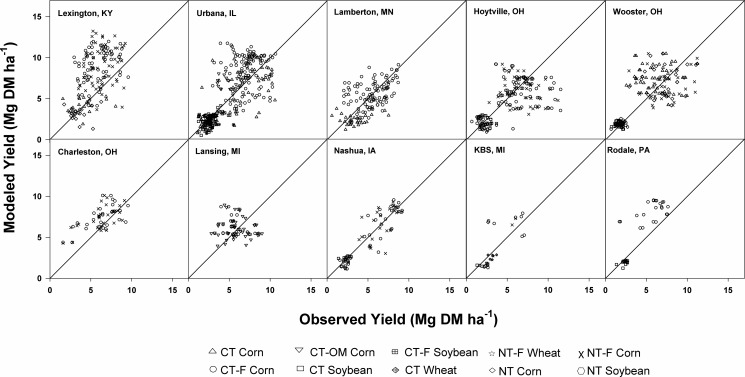
Comparison of CENTURY-modeled grain yields with observed yields at each experimental site. The grey lines are 1:1 lines. NT, CT, F, and OM indicate no tillage, conventional tillage, fertilization, and organic matter addition, respectively.

CENTURY overestimated average yields in many sites (Lexington, South Charleston, and Rodale and, to a lesser degree, Urbana and East Lansing) and that likely accounts for overestimation of SOC at some sites, but it underestimated yield in others (e.g. Hoytville) (Fig 4).

**Fig 5:**
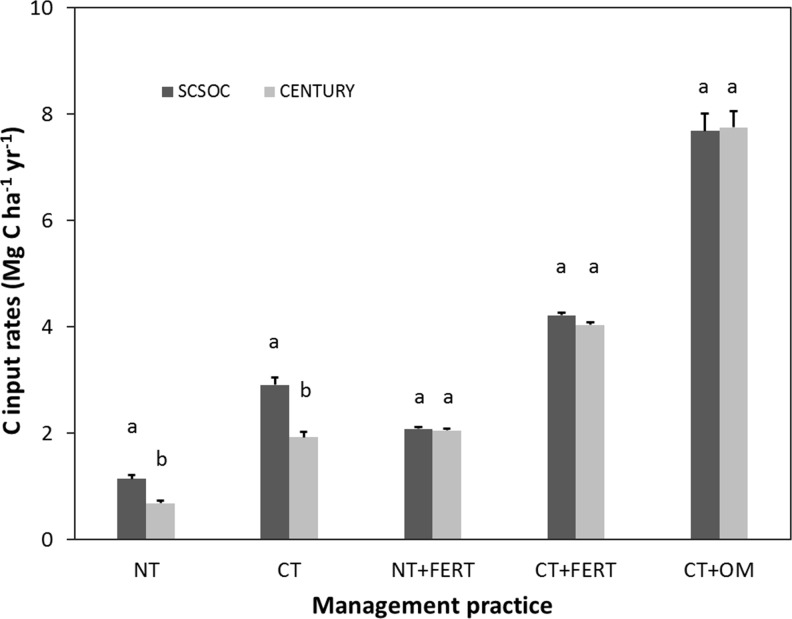
C input rates to soil under corn production (mean ± standard error). The rates were calculated by the plant growth module in CENTURY and agronomic indices coupled with observed yields in SCSOC, across management practices. NT, CT, FERT, and OM indicate no tillage, conventional tillage, fertilization, and organic matter addition, respectively. Based on Student’s t-test for paired data at *P<0*.*05*, different letters indicate that C input rates calculated by CENTURY and SCSOC are significantly different.

Also, CENTURY frequently underestimated observed yields and therefore C inputs in unfertilized systems, but this bias was not apparent at sites with synthetic fertilization or organic amendments (Fig 5).

**Fig 6:**
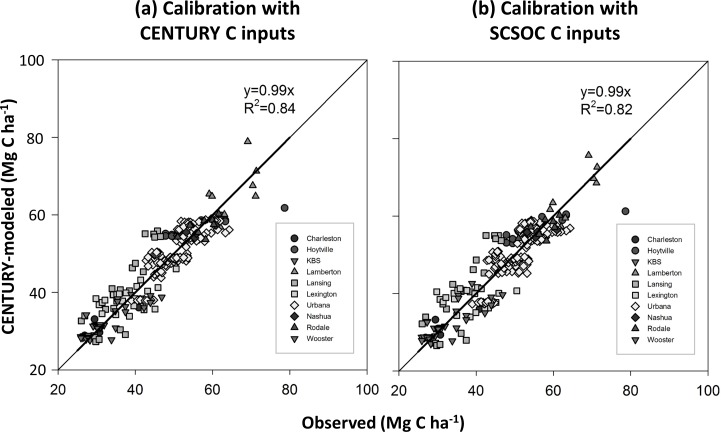
The results of calibration with (a) CENTURY-modeled C input rates and (b) yield-based C input rates. The grey lines are 1:1 lines while the black lines are regression lines. All *R*^*2*^ are significant at *P*<0.05.

Inverse modeling, notably improved the model fit. We estimated *f*_*S*_(0) by fitting the equations to multi-year SOC records and *mgmteff* in scenarios that used either CENTURY-modeled C input rates (*R*^*2*^ = 0.84, *P*<0.05) (Fig 6A) or yield-based C input rates (*R*^*2*^ = 0.82, *P*<0.05) (Fig 6B).

All parameter estimates were significantly different from one except for the *omeff* value that was estimated with observed yields (Table 3). Both the *ferteff* and *omeff* decreased when observed yields were used instead of CENTURY-based estimates to calibrate the SCSOC. This is likely due to the fact that observed C inputs in unfertilized plots were significantly higher than estimated values (Fig 5). There was no consistent difference between modeled and field-based estimates of C inputs in N fertilized systems regardless of fertilizer type or tillage. The C inputs were greatest in systems receiving organic inputs in addition to crop residues. Estimated C input rates to soil were always lower for fertilized plots under NT than CT because both CENTURY and SCSOC assume only 5% of aboveground residues are mixed with soils in NT systems and assume that 95% of C inputs are added under CT. This results in additions of ~2 Mg C ha^-1^ yr^-1^ of C under NT corn that is fertilized with N, which is about half the amount added under similarly managed CT (Fig 5). If one altered these assumptions to reflect shifts toward use of chisel plows instead of moldboard plowing in CT systems and account for the mixing action achieved by earthworms one might use factors closer to 0.85 and 0.15 in CT and NT systems respectively. Regardless of C placement assumptions, in all cases coefficients describing management effects on decay suggested there is a stimulatory effect of fertilizer addition on decay. The *ferteff* parameter, which was calibrated using data from all sites along with yield-based C input rates, was significantly different from one (*P<0*.*05*) (Table 3), implying that SOM decay rates occurring during the periods following fertilization were 1.24 (low N input rate) ~ 1.59 (high N input rate) times faster than that during unfertilized periods. By multiplying the *ferteff* coefficient with 1.4, the default value of *clteff*, we estimate the calibrated *mgmteff* would be 1.7 ~ 2.2.

**Table 3:** Parameters inversely estimated using the SCSOC. Parameter estimates, which were significantly different from zero (initial fraction of slow SOC) or unity (management effect) at *P*<0.05), are reported as mean ± standard error. NS indicates not significant from zero. Three levels of N fertilization were classified as low (≤100), mid (100~200), and high (≥200) kg N ha^-1^ rates; two levels of OM addition were classified as mid (≤10) and high (≥10) t dry matter ha^-1^ rates.

	C input rates based on	Parameters estimated									
		**_________________________________________Initial slow SOC pool (fraction of total) _________________________________________________**									

**Sites**		Lexington, KY	Urbana, IL	Lamberton, MN	Hoytville, OH	Wooster, OH	South Charleston, OH	East Lansing, MI	Nashua, IA	KBS, MI	Rodale, PA
	**Observed Yield**	0.60±0.04	0.36±0.030.66±0.020.36±0.020.56±0.02	0.78±0.03	0.50±0.04	0.70±0.06	0.49±0.12	0.66±0.09	0.43±0.08	0.61±0.14 0.65±0.16	0.35±0.07
	**CENTURY modeled**	0.54±0.04	0.27±0.040.50±0.020.19±0.020.40±0.02	0.69±0.03	0.35±0.03	0.51±0.05	0.35±0.03	0.51±0.05	0.27±0.06	0.46±0.130.47±0.14	0.29±0.06
		**_____________________________________________________Management effect (unitless)__________________________________________**									
	
**N inputs**		***ferteff***		***omeff***							
	**Observed Yield**	Low	1.24±0.09								
		Mid	1.37±0.11	Mid	1.07±0.16 (NS)						
		High	1.59±0.08	High	1.26±0.23 (NS)						
	**CENTURY modeled**	Low	1.34±0.11								
		Mid	1.74±0.16	Mid	2.10±0.28						
		High	2.19±0.13	High	2.02±0.38						

This is similar to what Kwon and Hudson [10] found using data from the Morrow Plots alone where they found *mgmteff* needed to be given a value of up to 2 for the year where N fertilizer was applied along with tillage. Further studies might need to explore relationships between SOC and *ferteff* using a larger data set to explore how fertilizer rate and form might interact with decay rates and/or improve existing stoichiometric controls for C transfers among the SOM pools that exist within CENTURY. In this work, the numbers of studies used to estimate coefficients for non-fertilized, low, medium, and high N inputs rates were relatively small.

Again the result for *omeff* in the directly measured scenario was greater than, but not significantly different from one (Table 3), which is consistent with the fact that effects on SOC might vary with manure type [29]. This would indicate that calibrating multiple *omeff* to describe feedbacks from the addition of a wide range of rates (4.5 ~ 18 Mg ha yr^-1^) and types (estimated C/N) of organic additions might be appropriate instead of using only two *omeff* coefficients to describe them all.

Recently, Fujita et al. [13] used inverse modeling to calibrate a coupled C and N based decomposition model and demonstrated that by accounting for the influence of recent management on the active fraction and stoichiometry of organic matter inputs they could improve decay model performance at larger temporal or global scales. The need for calibrating the slow pool (*f*_*S*_(0)) along with *mgmteff* also suggests that the effects of recent management on SOC dynamics should be incorporated into the analysis, and is typically addressed in CENTURY simulation modeling frameworks [e.g. 12]. Estimates for *f*_*S*_(0) that were based on observed yields ranged between 0.35 ~ 0.78 (Table 3); this agrees with model derived estimates of recalcitrant fraction pool-size reported in Falloon and Smith [30]. Positive effects of recent management on SOC is reflected by a shift of stocks from the recalcitrant to the slow pool. Compared to yield-based estimates of *fs*, estimates derived from CENTURY-modeled yield were consistently reduced by an average of 53% (decreases range from 17~176% based on comparison of initial and adjusted values). Generally, the sites where grass vegetation was present just prior to the start of the study period had higher initial *fs* values and SOC levels than sites with a history of continuous cultivation. The highest *f*_*S*_ (0) value (0.78) was estimated for Lamberton, followed by the Wooster (0.70), East Lansing (0.66), KBS (0.65), and Lexington (0.59) sites where grass vegetation was dominant prior to implementation of experimental treatments. One exception was the KBS site (*f*_*S*_ (0) = 0.60) but this estimate was relatively uncertain based on the site’s high standard error. Intermediate *f*_*S*_(0) values, about 0.50, were estimated for two Ohio sites (South Charleston and Hoytville) where cultivation had been conducted for less than 10 years. The *f*_*S*_(0) values estimated at Rodale and Nashua sites where cultivation had been conducted for at least 10 years ranged from 0.35 to 0.43 (Table 3). At the Urbana site, where practices had been in place for over 80 years prior to the starting year of simulation (1955), estimated *f*_*S*_(0) values varied significantly among treatments. All *f*_*S*_(0) were greater than 0.50 when treatments had a history of cultivation with OM addition while they were less than 0.40 without OM addition.

Estimates of *fs*(0) obtained using C inputs estimated by CENTURY also varied to reflect site history. These results suggest *f*_*S*_(0) values can be adjusted to account for soil condition during model initialization. It should be noted that site history is also reflected in estimates of *f*_*P*_(0) because values were determined by difference (*f*_*P*_(0) = 1–0.02—*f*_*S*_(0)) and so vary as a result of changes in *f*_*S*_(0). Estimates of *f*_*P*_(0) ranged from 0.37 ~ 0.63, and were higher for sites calibrated with CENTURY-modeled yield.

### Uncertainty analyses

**Fig 7:**
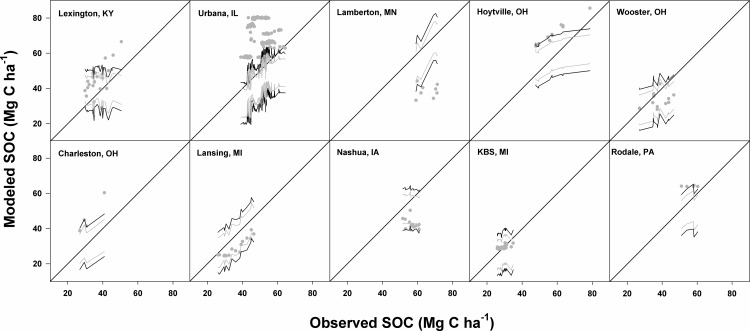
95% confidence intervals constructed by using 1,000 Monte Carlo (MC) simulations and parameter statistics. The statistics were derived from either inverse modeling of observed SOC with yield-based C input rates (grey solid line) or inverse modeling of “synthetic” SOC with “synthetic” yield-based C input rates (black solid line). Grey dots plot CENTURY-modeled SOC against observed SOC. The diagonal lines are 1:1 lines.

At three sites (i.e. Wooster, Lansing, and KBS) (Fig 7), CENTURY-modeled SOC fell within the 95% confidence intervals (CI) of estimated SOC that was derived from inverse modeling of observed SOC data followed by MC simulations.

Most model estimates for SOC at the Lexington site also fell within that CI. This implies that model calibration was not needed to obtain acceptable estimates at those 4 sites and that new parameter estimates will not improve model predictions statistically if uncertainties are considered. When uncertainties or errors of measurements related to observed SOC and yield data were further explored using inverse modeling of “synthetic” SOC and yield data coupled with MC simulations, wider CIs (black lines) that were produced encompassed some estimated SOC values at two sites (South Charleston and Nashua) but values remained outside the limits at 4 other locations. This means that model calibration is required to achieve acceptable estimates of SOC at those locations (Fig 7). Even after 95% CIs were constructed from inverse modeling of “synthetic” data, problems with over- (Urbana) and under- (Lamberton) estimation of SOC remained, indicating that site-specific calibration is needed to effectively model SOC at those sites. However, we should also recognize that we have assumed a ±10% variation with respect to the mean, which may not reflect the full variation in the samples.

### Improving model predictions

**Fig 8:**
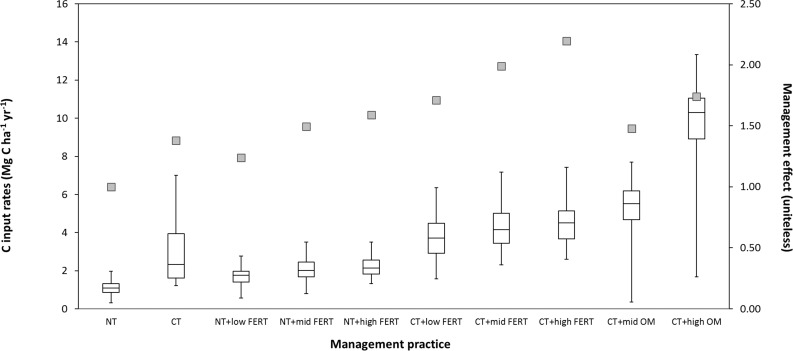
Relationships between management practices and C inputs under corn production. The C inputs were derived from observed yields and agronomic indices (box plot) or management effect estimated (line plot). NT, CT, FERT, and OM indicate no tillage, conventional tillage, fertilization, and organic matter addition, respectively. Three levels of N fertilization are classified as low (≤100), mid (100~200), and high (≥200) kg N ha^-1^ rates and two levels of OM addition as mid (≤10) and high (≥10) t dry matter ha^-1^ rates, respectively.

The need to calibrate one or more of the parameters that adjust decay rates might suggest that there is also a need to refine the mathematical functions associated with decay rates. Our finding, which suggests that components of *mgmteff* require adjustments, agree with previous work wherein individual CENTURY modelers relied on expert judgment to adjust this parameter. Importantly, we observed a positive relationship between *mgmteff* and C input rates (Fig 8); this effect was most clear when interactions with tillage and amendment type are considered.

This is consistent with the findings of Huggins et al. [31], who modeled SOC changes at Urbana using a one-compartment, first-order decay model, and Kwon and Hudson [10], who used the SCSOC to model data collected at the Urbana site. Saturation of SOC is sometimes used to explain rapid decay rates [32] but we discount this explanation, as increases in turnover observed within individual site comparisons are similar or greater in soils with lower SOC levels.

The SOM decay process is sensitive to both the mass of SOM and the characteristics of the microbial community (e.g. size and activity), which are also influenced by N fertilization history [33], rather than simply the mass of SOM as assumed in many SOM models, including CENTURY. Stimulation of soil microbial activity caused by increased C inputs can result from alleviation of C limitation. Stimulation of microbial activity can also result from alleviation of N limitations through fertilization that induces production of enzymes to degrade SOC [34]. The importance of these interactions is apparent in recent SOM models that explicitly incorporate microbial population size/activity (e.g. [35, 36]). Positive priming by C and N additions that are known to occur [37], are not effectively captured by established process models. Feedbacks of N additions were captured in this work through statistical adjustment of rate modifying coefficients. It is possible that the feedback of C additions could be achieved by replacing the first-order kinetic equation used to describe SOC decay with a nonlinear Michaelis-Menten rate equation to better describe the relationship between C inputs and microbial activity. Reliance on such detailed formulations, however, would make model parameterization more complex [36].

For example, if we replace *mgmteff* with a nonlinear Michaelis-Menten rate equation and a metabolic residue pool (*C*_*MR*_) in order to represent the microbial activity incorporated into the SOM decay rate calculations for the active and slow pools, we can redefine a site-specific decay rate coefficient for SOM pool *j* as follows:

$$k_{j}^{*}(m)=k_{j}^{0}\cdot defac(m)\cdot \left(\frac{V_{\max}\cdot C_{MR}(m)}{K_{m}+C_{MR}(m)}\right)\cdot C_{j}(m)$$ (8)

Where $k_{j}^{0}$ is the maximum decay rate coefficient of SOM pool *j* in uncultivated soil, *V*_*max*_ represents maximum microbial activity at saturating substrate concentrations and Michaelis constant *K*_*m*_ is the substrate concentration at which the reaction rate is half of *V*_*max*_.

When we employed this equation to estimate the active and slow pools, we obtained a relationship between observed and modeled SOC that was similar to that obtained after calibration of *mgmteff* (*R*^*2*^ = 0.86, *P*<0.05). These findings suggest use of alternative rate laws could significantly improve models of both long-term and short-term C dynamics.

## Conclusion

Drawing on data from multiple sites, we were able to generate robust parameter estimates using climatic factors and C input rates derived from CENTURY outputs simulated by Ogle et al. [11] when they were combined with yield-based estimates of C inputs. A surrogate CENTURY model (SCSOC) was used to calculate time-series for SOC stocks and optimize the fit to observed SOC by adjusting the coefficient that determines management effects on SOC decay rates.

Inverse modeling results suggested N additions accelerated SOC decay for the year following application up to 1.5 fold when the model was driven using yield-based C inputs, or by 2 fold when CENTURY-modeled C inputs were used. In addition, we found SOM decay rates were positively related to the magnitude of C inputs to the soil, and this indicates that the structure of CENTURY may need some revision to improve the estimation of production. Our evaluation suggests SOM decay may be better represented as Michaelis-Menten kinetics than first-order kinetics. This dependence of turnover times on C and N input rates indicates that C and N priming has significant implications for attempts to manage soil C levels and should be explicitly represented in SOC models. Finally, calibration of the initial fraction of SOC in the slow SOM pool against SOC levels allowed us to account for site history, suggesting that values should be adjusted to account for soil condition during model initialization. Further calibration efforts are needed to describe feedbacks from C inputs made in various forms over a wider range of rates.

## Supporting information

S1 Table Default values of maximum decay rate coefficients used in CENTURY and SCSOC. (DOCX)

S1 File Data used for inverse modeling. (XLSX)
