# Correction: Use of inverse modeling to evaluate CENTURY-predictions for soil carbon sequestration in US rain-fed corn production systems

**DOI:** 10.1371/journal.pone.0173729

**Published:** 2017-03-06

**Authors:** Hoyoung Kwon, Carmen M. Ugarte, Stephen M. Ogle, Stephen A. Williams, Michelle M. Wander

Table 3 appears incorrectly formatted in the published article. Please see the correct Table 3 and its caption here.

**Table 3:** Parameters inversely estimated using the SCSOC. Parameter estimates, which were significantly different from zero (initial fraction of slow SOC) or unity (management effect) at *P*<0.05), are reported as mean ± standard error. NS indicates not significant from zero. Three levels of N fertilization were classified as low (≤100), mid (100~200), and high (≥200) kg N ha^-1^ rates; two levels of OM addition were classified as mid (≤10) and high (≥10) t dry matter ha^-1^ rates.

	C input rates based on	Parameters estimated									
		**_______________________________________Initial slow SOC pool (fraction of total) _____________________________________**									
**Sites**		Lexington, KY	Urbana, IL	Lamberton, MN	Hoytville, OH	Wooster, OH	South Charleston, OH	East Lansing, MI	Nashua, IA	KBS, MI	Rodale, PA
	**Observed Yield**	0.60±0.04	0.36±0.03	0.78±0.03	0.50±0.04	0.70±0.06	0.49±0.12	0.66±0.09	0.43±0.08	0.61±0.14	0.35±0.07
			0.66±0.02							0.65±0.16	
			0.36±0.02								
			0.56±0.02								
	**CENTURY modeled**	0.54±0.04	0.27±0.04	0.69±0.03	0.35±0.03	0.51±0.05	0.35±0.03	0.51±0.05	0.27±0.06	0.46±0.13	0.29±0.06
			0.50±0.02							0.47±0.14	
			0.19±0.02								
			0.40±0.02								
		**_______________________________________Management effect (unitless)___________________________________________**									
**N inputs**		***ferteff***		***omeff***							
	**Observed Yield**	Low	1.24±0.09								
		Mid	1.37±0.11	Mid	1.07±0.16 (NS)						
		High	1.59±0.08	High	1.26±0.23 (NS)						
	**CENTURY modeled**	Low	1.34±0.11								
		Mid	1.74±0.16	Mid	2.10±0.28						
		High	2.19±0.13	High	2.02±0.38						
