# Supplementary material for: Use of inverse modeling to evaluate CENTURY-predictions for soil carbon sequestration in US rain-fed corn production systems
Source: PLoS One. 2017 Feb 24;12(2):e0172861. doi: 10.1371/journal.pone.0172861 (PMC5325579; doi:10.1371/journal.pone.0172861)
Supplement: S1 Table — (DOCX) [file pone.0172861.s001.docx]

**S1 Table. Default values of maximum decay rate coefficients used in CENTURY and SCSOC**

| **Pool** | **Rate (day^-1^)** |
| --- | --- |
| Structural surface litter | 0.01068 |
| Metabolic surface litter | 0.04055 |
| Structural soil litter | 0.01342 |
| Metabolic soil litter | 0.05068 |
| Surface active | 0.01644 |
| Soil active | 0.02000 |
| Soil slow | 0.000548 |
| Soil passive | 0.000012 |
